# Use of Photo-Identification and Mark-Recapture Methodology to Assess Basking Shark (*Cetorhinus maximus*) Populations

**DOI:** 10.1371/journal.pone.0150160

**Published:** 2016-03-01

**Authors:** Mauvis A. Gore, Peter H. Frey, Rupert F. Ormond, Holly Allan, Gabriella Gilkes

**Affiliations:** 1 Marine Conservation International, South Queensferry, Edinburgh, EH30 9WN, United Kingdom; 2 Heriot-Watt University, Edinburgh, EH14 4AS, United Kingdom; 3 University of Plymouth, Plymouth, PL4 8AA, United Kingdom; University of Illinois at Chicago, UNITED STATES

## Abstract

Following centuries of exploitation, basking sharks (*Cetorhinus maximus*) are considered by IUCN as Endangered in the Northeast Atlantic, where they have now been substantially protected for over two decades. However, the present size of this population remains unknown. We investigated the use of photo-identification of individuals’ dorsal fins, combined with mark-recapture methodology, to investigate the size of populations of basking shark within the west coast of Scotland. From a total of 921 encounters photographed between 2004 and 2011, 710 sharks were found to be individually identifiable based on dorsal fin damage and natural features. Of these, only 41 individuals were re-sighted, most commonly both within days of, and close to the site of, the initial encounter. A smaller number were re-sighted after longer periods of up to two years. A comparison of the distinguishing features of individuals on first recording and subsequent re-sighting showed that in almost all cases these features remained little changed, suggesting the low re-sighting rate was not due to a loss of distinguishing features. Because of the low number of re-sighting we were not able to produce reliable estimates for the long-term regional population. However, for one 50 km diameter study area between the islands of Mull, Coll and Tiree, we were able to generate closed-population estimates for 6–9 day periods in 2010 of 985 (95% CI = 494–1683), and in 2011 of 201 (95% CI = 143–340). For the same 2011 period an open-population model generated a similar estimate of 213 (95% CI = 111–317). Otherwise the low rate and temporal patterning of re-sightings support the view that such local basking shark populations are temporary, dynamic groupings of individuals drawn from a much larger regional population than previously supposed. The study demonstrated the feasibility and limitations of photo-identification as a non-invasive technique for identifying individual basking sharks.

## Introduction

With sharks gaining recognition as a global conservation priority, a wider range of methods for assessing population levels is urgently required to underpin management and protection of the most vulnerable species. Basking sharks (*Cetorhinus maximus*) are the second largest species of fish globally and have been subject to commercial exploitation in the North Atlantic for over 200 years [1] [2] [3]. Following concern over their apparent decline, they were given full protection in British territorial waters in the 1980s, and subsequently in waters of some other countries bordering the North Atlantic and Mediterranean [3]. They have also been listed in Appendix II of the Bern Convention, Appendix II of CITES, and Appendix 1 of CMS. In the IUCN Red List they are assessed as globally Vulnerable, but both North Pacific and Northeast Atlantic stocks are considered Endangered [3].

Despite this conservation concern, no scientific estimate of basking shark population size has yet been possible for any region within their circumglobal distribution [4]. This is for several reasons. First, for most of the year they move undetected through deep water, being observed only when surface-feeding in locations where zooplankton concentrate near the surface [5]. Second, as data from satellite tags indicate ([6] & Gore et al. unpublished data) [7], usually only a small and quite variable proportion of the animals present in an area are at or near the surface at any one time; this makes the results of boat-based visual surveys and public sighting schemes of limited value in estimating true population size. Third, as also indicated by satellite tag data [6] [8], basking sharks appear to move relatively frequently over large distances between different foraging areas, making tracking and monitoring their numbers by one or a few boats impracticable. Fourth, this same behaviour, combined with the relatively high turbidity of the waters where they occur, makes underwater visual surveys by divers, such as those undertaken for reef shark species [9] [10], impracticable. Finally, historic fisheries records for basking shark, as well as other large shark species, are of limited value for estimating present-day abundance, not least because of the problem of obtaining present-day catch data for stocks that are now scarce or protected [11].

The only published indication of potential basking shark population size is an analysis by Hoelzel et al. [12] of mitochondrial DNA samples obtained from areas in the North Atlantic and South Pacific. They found little genetic differentiation between global regions, leading them to suggest an effective global population size (*N*_*e*_) of as low as 8,200, perhaps consequent to a genetic bottleneck during the recent past. However, recent satellite tracking studies have revealed transoceanic and transequatorial migrations of individual basking sharks [6] [7], suggesting a degree of continued genetic mixing that offers an alternative explanation for this low genetic diversity. Given the gaps that remain in our understanding of basking shark movements and population structure, as well as the impracticality of employing catch per unit effort (CPUE) visual surveys or fishery-dependent population assessment methods [4], it became urgent to explore alternative approaches for estimating basking shark population size.

Mark-recapture methods have been used to estimate population abundance for several species of shark [13] [14] [15] [16] [17], but the method had yet to be applied to the basking shark. For fish species, mark-recapture techniques generally involve the physical attachment of tags that allow individuals to be recognized upon recapture [18]. However, tagging can be logistically difficult for species that are hard to capture or relocate, and long-term studies have been compromised by low tag retention rates in some sharks, including the whale shark [17] [19]. Photo-identification offers a non-invasive alternative for populations in which a reasonable proportion of individuals can be recognized on a recurring basis as a result of natural markings and features [20]. Unlike tagging or artificial marking, photo-identification allows the ‘capture’ and ‘recapture’ (re-sighting) of individuals without inducing stress that may influence natural behaviour or present a safety hazard. Photo-identification is relatively cost-effective and allows the participation of non-specialists in the data collection process [21]. There is a risk of making false positive or negative identifications, especially if due care is not taken, since different individuals may appear similar or markings change over time [16]. Marshall and Pierce [22] provide a useful overview of some of the issues that need to be considered in applying photo-identification methods to sharks and rays.

To date, mark-recapture techniques using underwater photographs taken of body patterns and fins have been successfully used to estimate regional populations of white sharks (*Carcharodon carcharias)* [23], whale sharks (*Rhincodon typus*) [15] [16] [17], and nurse sharks (*Ginglymostoma cirratum*) [13]. In addition, white shark dorsal fins have been photographed when the sharks cruised at the surface [14] [24] and the data used to derive population estimates [25]. These studies have demonstrated that in some elasmobranch aggregations, the longevity of individually recognisable features and the degree of individual site-fidelity are sufficient to allow both long-term recognition of individuals and the estimation of population abundance using mark-recapture models.

To a very limited degree, other researchers have attempted to use photo-identification to re-identify individual basking sharks. A re-sighting of an individual has been reported by Sims et al. [26], while Darling and Keogh [27] used dorsal fin photos to identify individual sharks during a three-week field study on the movements and behaviours of a small population observed in British Columbia, Canada. Given the need to assess the current status of basking sharks and the additional value of re-sightings for illustrating the extent of shark movements in comparison with satellite tag data ([4] & Gore et al. unpublished data), we sought to determine whether basking sharks could be individually recognised over time from digital images of their dorsal fins.

The west coast of Scotland has seasonal sightings of basking sharks, with the Inner Hebrides and Firth of Clyde regions recognised as hotspots where surface sightings are relatively common between June and October [6] [8] [28] [29]. Having undertaken boat-based surveys of both these regions on an annual basis since 2004, we considered that the aggregations of surface-feeding basking sharks that we had found occurring regularly in these areas might offer an opportunity to trial photo-identification and cataloguing of individual sharks. Accordingly, a study was undertaken to evaluate the viability of photo-identification for this species and to test the potential for using mark-recapture methods for generating estimates of local or regional basking shark populations. We found that a majority of individuals had sufficiently distinct dorsal fins that photo-identification could be used to recognise therm. The rate of re-sighting was low, but enabled reliable estimates to be generated of the size of local populations over short time periods.

## Materials and Methods

### Photograph collection and preparation

**Fig 1:**
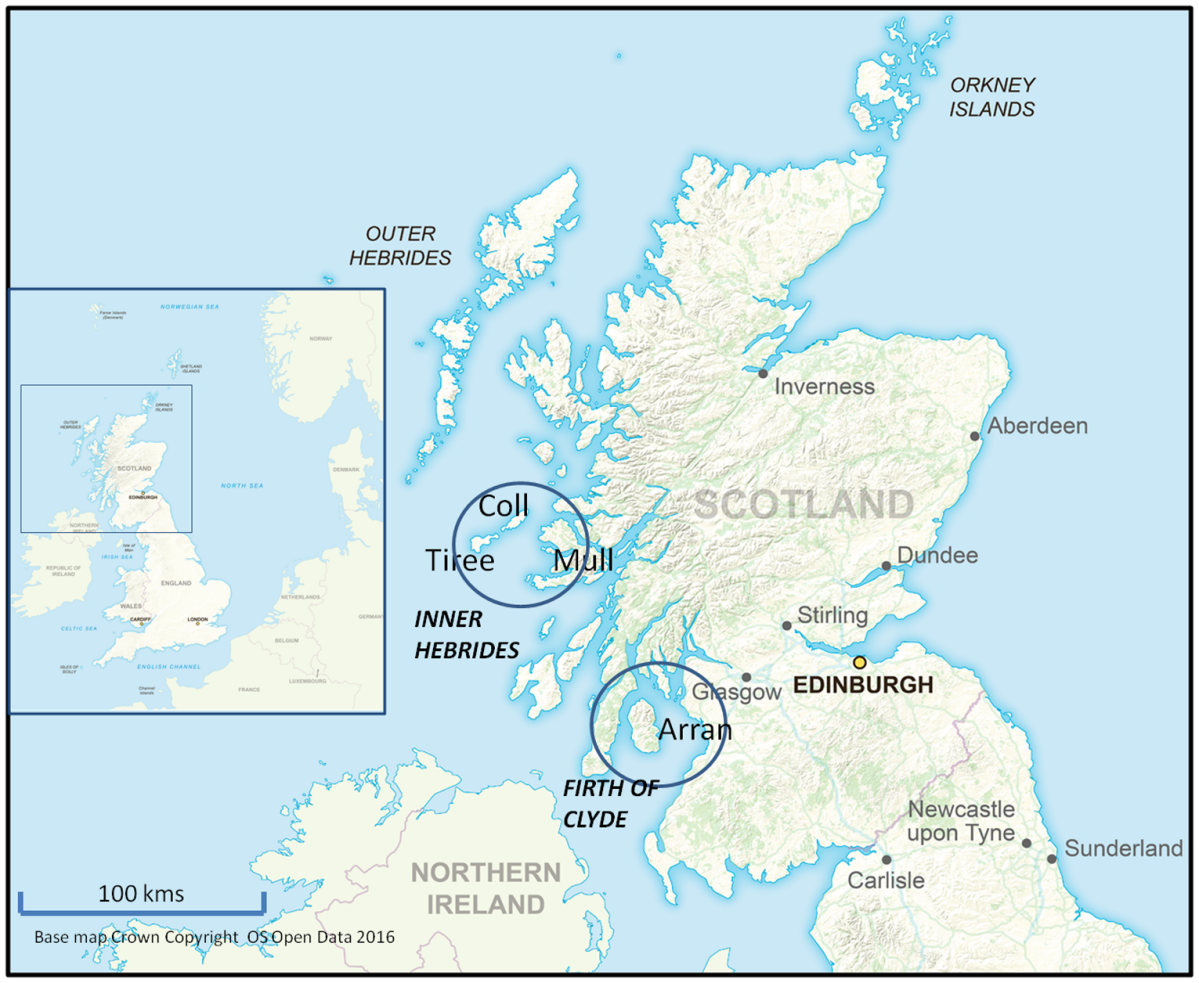
The study areas in relation to the west coast of Scotland. The main part of the figure shows the locations in relation to the rest of Scotland of the two study areas (a) the Inner Hebrides study area (including the Isles of Mull, Coll and Tiree) and (b) the Firth of Clyde study area, including the Isle of Arran. The inset shows the location of Scotland in relation to the coast of North-west Europe. (Base map Crown Copyright OS OpenData 2016.)

Digital images of basking shark first dorsal fins (dorsal fins) were taken off the west coast of Scotland from 2004 to 2011 during a series of summer to autumn surveys averaging 25 days per year. These boat-based surveys used a 6.4 m craft, along with vessels of opportunity, to locate and photograph surface feeding basking sharks within two study areas—the Firth of Clyde and a portion of the Inner Hebrides that included the west coast of the Isle of Mull and the Isles of Coll and Tiree—each about 50 km in diameter (Fig 1). Images were captured using a Canon EOS 350D Digital SLR Camera with Canon zoom lens (70–200 mm, 1:2.8 image stabilised) and a Canon 1.4x extender lens. Photographs were taken as high quality JPEG colour images rather than RAW as a compromise between quality, storage capacity available and handling time. Where possible at least 12–20 photographs were taken of each new individual shark both to allow for glare or potential distortion of the fin in the image, and to ensure optimal lighting for revealing the details of lesions, marks and pigmentation. So far as was possible, photographs were taken of both sides from a distance of 10 m or less and when at least 60% of the fin was showing above water. The aim was to obtain at least three well-illuminated photographs of each side of the dorsal fin of each shark encountered.

For each encounter the GPS coordinates, time and sea state were recorded, along with notes on behaviour and group size. Total length estimates were made using the length of the vessel, which has markings at 1 m intervals, as a visual reference. To avoid disturbance to the shark, the boat did not normally approach closer than 10m, although subsequently sharks often came alongside while the boat was stationary. The work was carried out under licence to the UK Home Office and Scottish Natural Heritage during satellite tagging work. A limited number of additional photos were submitted by a range of private vessel operators, research organizations, and members of the public. Photographs were assessed for quality as suggested by Auger-Méthé & Whitehead [30].

Specifications for the quality and type of photographs required to permit matching and data analysis were developed by MG and then discussed at a workshop during the “Basking Sharks—A Global Perspective” conference held in the Isle of Man in 2009. The specifications have since been published in a booklet funded by the present project [31]. The photos were catalogued into tiered digital folders according to year, date, individual shark identification number and time of encounter.

### Cartoons and classification

**Fig 2:**
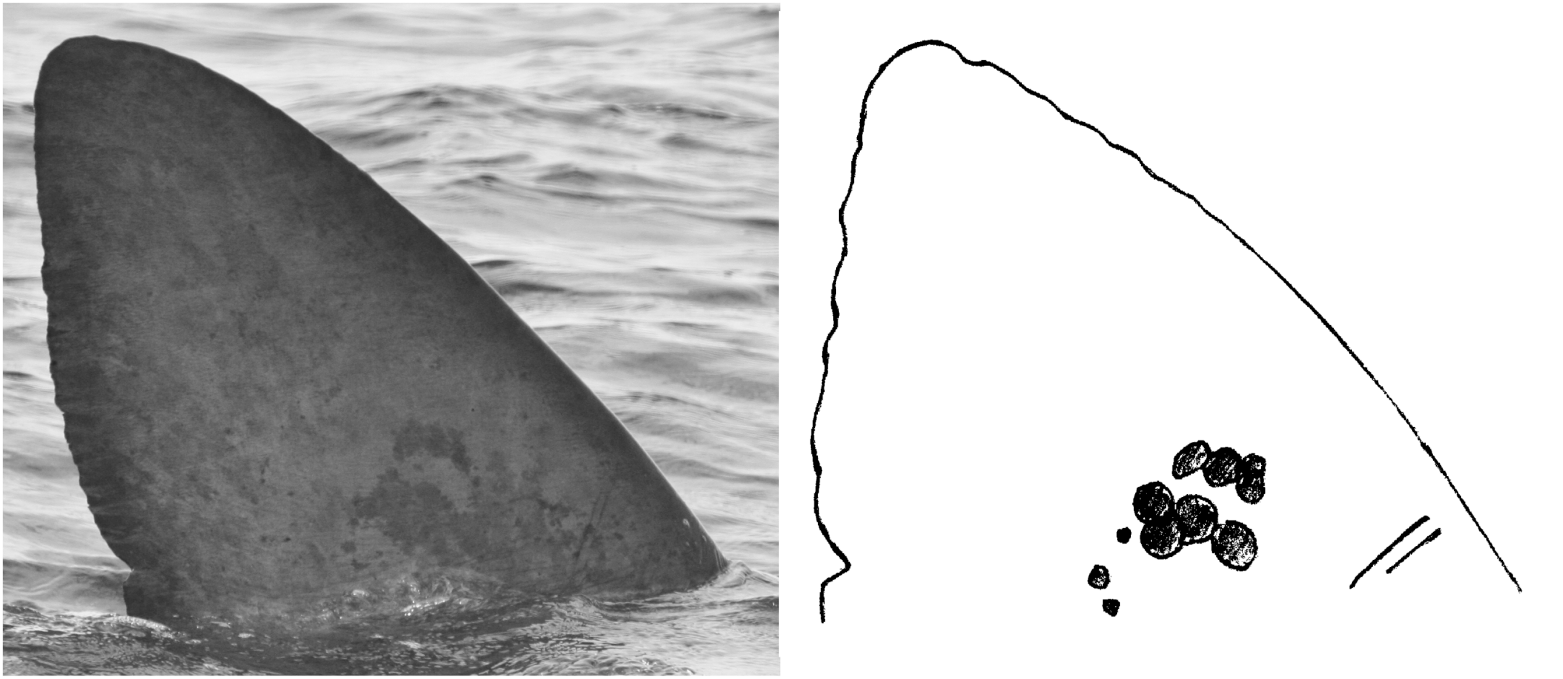
Sample basking shark dorsal fin photograph and sketched cartoon. The figure illustrates how a dorsal fin photograph is used to generate a cartoon (sketch diagram) to aid recording and cataloguing of distinctive features for individual identification.

Based on examination of all the photographs from each encounter, standardised sketches (cartoons) of both sides of the dorsal fin of each shark were created to assist in the recognition of individuals. Fin profiles (the shape) were sketched or traced from a computer screen and the main surface features added, including injuries, scars, parasitic copepods, epizoic barnacles and pigmentation marks (Fig 2). A total of 1,842 cartoons of both right and left sides of the dorsal fin were produced, based on 921 basking shark encounters that, within the study areas, between 2004 and 2011, resulted in photographs of sufficient quality. Besides being a convenient tool for fin comparison, the process allowed researchers to gain visual familiarity with the individual sharks photographed over the study period.

With the aid of the cartoons, the dorsal fin characteristics of each individual shark were classified in a computer database that included fields for recording fin shapes, distinctive markings and patterning, and scars and injuries (S1 Table). Identifiable dorsal fin characteristics were classed as either natural features (such as the shape of the leading edge) or as damage (for example a notch in the trailing edge). Damage was further distinguished according to whether it was to the edge of the fin or to its surface. The resulting database matrix provided a systematic means by which individual sharks with similar fin attributes could be segregated and then compared.

**Fig 3:**
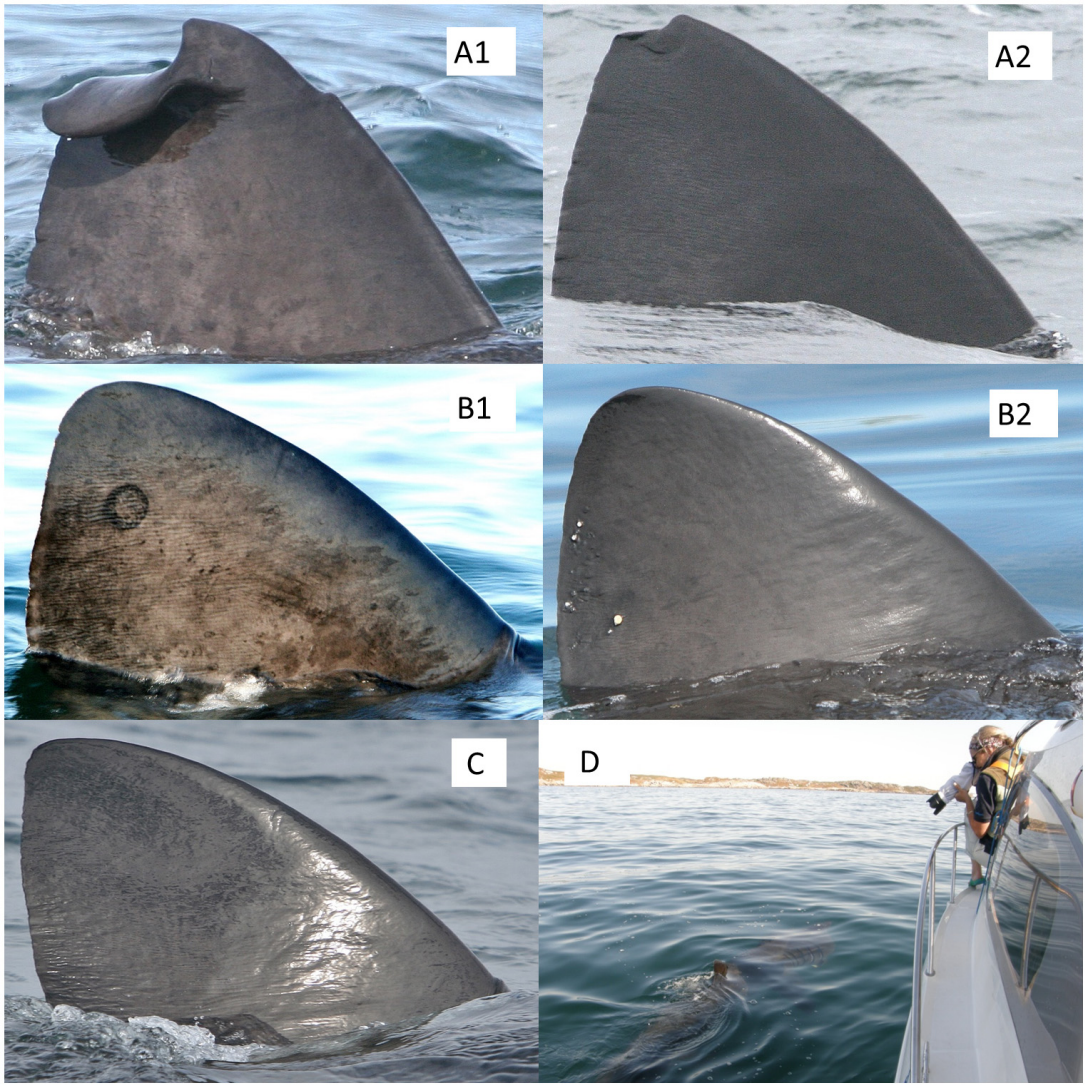
Sample photographs of fins assigned to each distinctiveness grade. A1—very conspicuous marks or injuries, likely to be recognisable in the very long term (a badly damaged fin apex), A2—clear marks or injuries, likely recognisable across year (a smaller injury to the fin apex), B1 -surface features or patterns such as lamprey scars and mottling—likely recognisable during a season (a conspicuous lamprey scar), B2—less permanent surface features, such as copepods, likely recognisable only in the short-term (note the clusters of copepods to the rear of the fin), and C—no readily evident distinguishing marks or features (although some marking and crenulation is still visible around the lower rear margin). Image D shows the situation in which quality images were typically obtained, with a surface feeding shark approaching close to a stationary boat.

Importantly, each fin was also graded according to its degree of distinctiveness, which is the ease with which it might be recognised again (Fig 3). The most distinctive fins (grade A) were defined as those with conspicuous marks or severe injuries occupying at least 5% of the fin surface area. Those that had features that seemed certain to make them recognisable across years, even in photographs of only moderate quality, were graded A1. Fins that were slightly less distinctive were classified as grade A2. Fins which had only finer scale marks or injuries occupying <5% of the surface area were divided between those where likely permanent patterns or marks were evident (grade B1) and those where the most distinctive features were attached copepods or barnacles or small cuts in the fin edge, that might disappear in the medium term (grade B2). Finally, those fins lacking any distinct visible features, and so potentially unrecognisable, even if photographed again within a very short period of days, were classified as grade C.

The key distinction for the present analysis was between the relatively small proportion of C grade individuals, which could not be considered as being marked for mark-recapture modelling purposes, and the majority which were potentially re-identifiable and so could be considered as having been marked. The distinction between A1, A2, B1 and B2 grades was not used in making population estimates in the present study, but was used to assess the longevity of the different types and scale of fin features, in case the evidence indicated that less conspicuous features became undetectable within the timeframe of the analyses. In the event our photographic data demonstrated that with rare exceptions distinguishing features remained discernible both within a summer season, and between years (see Results). This conclusion is supported by comparable studies on other species e.g. white shark, in which patterns of fin edge damage remain distinguishable for up to twenty years (see Discussion). Thus, for the within season analyses presented here, the re-sightings of all sharks in grades A1 to B2 were pooled. The maximum period between initial sighting and re-sighting used in these analyses was 14 days, while the minimum interval between encounters with the same shark that was recorded as a re-sighting for the purpose of data analysis was 2 h. Re-sightings within < 2 h were due to unintentional re-documenting of a shark before the survey vessel had moved away from the area where it was first recorded. If there were many sharks in the vicinity of the boat (sometimes 10 or more) it was not always possible to be sure at the time which ones had already been photographed, and which had not, but such duplicate records were normally easy to match up and exclude on subsequent laboratory study of the images.

### Identification of matches

Searching for and confirming matches between different sets of photographs for each new encounter (in order to determine when an individual had been re-sighted) was accomplished by a) systematically sorting the database by one or more attributes to look for similar individuals, followed by b) visual comparison of the respective cartoons, and finally by c) careful side-by-side examination of the relevant photographs. Following initial discovery, suspected matches were independently confirmed or rejected by a second experienced researcher. To avoid false positive matches that could confound mark-recapture analysis, a high level of certainty was required. Contrary to initial expectations, it was found that in most cases of confirmed matches the identity of the individual shark could be verified with a high degree of certainty.

With a view to semi-automating the process, trials were undertaken using Finscan software [32], originally developed as a means of matching images of cetacean dorsal fins. To compare the effectiveness of manual and computer-aided matching of basking shark fins, four individual researchers were given a series of quality photographs composed of ten unlabelled pairs of images (each pair being taken of the same shark on the same occasion) and asked to identify the matches using both methods. Whereas manual matching was completed by all researchers to 100% accuracy on all save B2 and C grade fins, the maximum achieved using Finscan was only 28%.

### Mark-recapture analysis

Following the verification of all re-sightings, methods for deriving mark-recapture abundance estimates from the dataset were assessed. Although the original intent was to apply models over a range of temporal scales, the relatively limited number of re-sightings restricted application of full mark-recapture analysis to data from two short sampling periods of 6–9 days within which sufficient numbers of re-sightings were obtained. These occurred during August–September 2010 and August 2011, both within the Inner Hebrides study area (Fig 1). For estimates of abundance, an open-population Jolly-Seber (JS) model would seem most likely to be applicable, allowing for population entries and departures throughout the sampling period [33]. However, over the short periods referred to above, the seasonal aggregations observed appeared temporarily stable and it seemed likely that relatively few individuals could have moved far enough away to leave the survey area altogether. Thus it seemed possible that, within the short time-frames concerned, the groupings of individuals might conform sufficiently to the assumption of demographic closure [34] for closed-population models also to be applied.

### Open-population models

Open-population models rely on individual capture histories to generate estimates and also require a relatively high number of individuals to be captured on more than one occasion to produce statistically robust outputs [35]. In this study, only the approximately week-long peak-sighting period in 2011 resulted in a concentration of re-sightings approaching a level that would allow for a statistically sound abundance estimate using a JS open-population model. While limited in its scope and reliability, this estimate nonetheless offered insights into the size and structure of basking shark aggregations in the Inner Hebrides study area. The JS model assumes that marks are permanent and never overlooked, that all individuals present in a population on a given sampling occasion (whether newly sighted or re-sighted) have an equal probability of capture and an equal probability of survival to the next sampling event, and that all emigration is permanent [36]. While these conditions are rarely if ever met fully in wild populations, our comparison of images obtained on initial sightings with those obtained on re-sighting indicates that only rarely will the fin features have changed sufficiently to make the individual unrecognisable after so short a period.

Four variants of the JS model were applied to this 2011 peak-sighting period dataset using the POPAN option within the program MARK [37]. The general model produced occasion-specific (t) estimates of p (probability of capture for an individual remaining in the study population), phi (apparent probability of survival, or the likelihood of an individual remaining within the study area), and pent (probability of entry into the study population). The three additional models featured one or more of these parameters specified as constant throughout the sample period. Most relevant to the current study, each model generated an estimate of population abundance for the sample period (*N*), defined as the total number of animals available to enter the study population. The goodness-of-fit of the general model to the data set was tested using the RELEASE program [38], with the number of parameters for each model adjusted to account for unidentifiable parameters resulting from the low rate of recapture. The goodness-of-fit test in program RELEASE was used in preference to those in the more current program MARK, because the latter involves more derived inputs and did not function satisfactorily with the small number of recaptures in the present dataset. To derive the best population estimate based on the four variants of the JS model, model comparisons and model-averaged estimates of *N* were made using Akaike Information Criterion corrected for small sample sizes (AICc) [39].

A further problem with estimating the total population of basking sharks from our data is that if only the recorded resightings are input into a model to derive an abundance estimates, this estimate will reflect only the portion of basking sharks in the population that carried natural markings or injuries and so were individually recognisable. Sharks that were naturally unmarked could not be recorded as re-sighted and so their numbers could not be directly estimated. To allow for the numbers of unrecognisable (grade C) sharks, the model outputs were interpolated by dividing the *N* values by the proportion of individuals (*θ*) adjudged unidentifiable (i.e. = numbers of A1+A2+B1+B2 grades observed / total number of individuals observed). This was done on a seasonal basis so as to provide an estimate of total population (*N*_*tot*_) (i.e. including both recognisable and non-recognisable individuals). In principle *θ* should be adjusted to allow for the fact that among the unmarked individuals observed there should also have been some undetected re-sightings, at a similar rate to that of marked individuals. However, given the very low rate of re-sighting among the marked individuals, it was decided to avoid this further correction of uncertain validity. There was however no reason to consider grade C sharks less likely to be captured, since all sharks encountered on the surface were photographed systematically, irrespective of whether they bore distinctive features or not, while the grading was only determined on subsequent inspection of the images.

Variance and standard error were extrapolated to the estimated total population (*N*_*tot*_) using the delta method (Table 1) [40]. Confidence limits for *N*_*tot*_ were assumed to occur with the same number of standard errors on either side of *N*_*tot*_ as for the corresponding *N* confidence limits [40].

**Table 1:** Equations for the Delta method and for the two closed-population models.

Delta Method	$Var(N_{tot})=N_{tot}^{2}(\frac{\text{var }N}{N^{{}^{{}_{2}}}}/+\frac{1-\theta }{n\theta })$
Schnabel’s Binomial	$N=\frac{\Sigma_{i=2}^{t}C_{i}M_{i}}{\Sigma_{i=2}^{t}R_{i}+1}$
Schumacher & Eschmeyer	$N=\frac{\Sigma_{i}^{t}C_{i}M_{i}^{2}}{\Sigma_{i=2}^{t}R_{i}M_{i}}$

*N*_*tot*_ = estimated total population present during the sample period (including grade C sharks), *N* = estimated recognisable population, *θ* = proportion of recognisable individuals in population, *n* = total number of individuals captured during the sample period, *C*_*i*_ = number of potentially recognisable individuals captured at occasion *i*, *M*_*i*_ = number of previously sighted, recognisable (‘marked’) individuals in the population immediately before sample *i* was taken, *R*_*i*_ = the number of re-sighted individuals in sample *i*, and *t* = the number of sample occasions in the sample period.

### Closed-population models

To provide a basis for comparison with the short-term JS estimates and to estimate local abundance over entire sampling seasons, closed-population models with varying sensitivity to violations of underlying assumptions were also applied. These models make similar assumptions to the open-population models save that the population is assumed to remain closed throughout the sampling period, with no new individuals joining it and none leaving [35]. While the assumption of demographic closure must be increasingly violated with increasing time for the 50 km diam Inner Hebrides study area (Fig 1), the reduced data requirements for these models compared to the open-population JS model made them a useful, additional means of further investigating the structure of the Inner Hebrides populations. The closed-population model options available within MARK were found unsuitable because they require individual capture histories, which proved limiting in the present case where only a few animals were re-sighted on more than one occasion. Therefore two simple models were applied with the relevant calculations completed by hand so as to provide for greater transparency.

The first closed-population model was a modified version of Schnabel’s binomial model (Table 1) [35]. This model is less biased for small sample sizes than the original Schnabel equation, although it remains highly sensitive to violations of the assumptions of equal catchability and population closure [35]. Variance was calculated as in Chapman [41], and due to a low number of re-sightings (*m* < 50), Chapman’s Poisson distribution table used to obtain the smallest 95% confidence intervals [42].

The second model was Schumacher & Eschmeyer’s regression (Table 1) [43]. This model is more robust to violations of underlying assumptions than the binomial model and can be graphed to demonstrate a goodness-of-fit to the dataset [35]. The best 95% confidence interval and variance were calculated following De Lury [44] and Ricker [42]. As with estimates from open population models, to allow for the numbers of unmarked sharks, values of *N* were divided by *θ*, the proportion of sharks graded as having individually recognisable fins (grades A1 –B2). Standard error and confidence intervals for *N*_*tot*_ were achieved using the delta method (Table 1) [40].

### Ethics Statement & Deposition of Data

The work described here was conducted under a licence (No. 11582) to work on a protected species issued by Scottish Natural Heritage and under licences to use animals in scientific procedures (PCW60/9071 and PIL 30/6819) issued by the United Kingdom Home Office in accordance with the Animals (Scientific Procedures) Act of 1986. All work was carried out in conformity with the relevant legislation and guidelines issued by these agencies. The boat used in photography did not approach closer than 10 metres to any animal, although these often themselves swam closer, and in no case was feeding behaviour disrupted by the work described here. All data and photographs on which this manuscript is based have been deposited in the Basking Shark Photo-identification Database established by the Shark Trust, Plymouth UK, and may be accessed via its dedicated website www.baskingshark.org.

## Results

### Survey Effort

Table 2 shows the distances covered and the duration of dedicated boat surveys undertaken in the Firth of Clyde and the Inner Hebrides study areas between May 2004 and August 2011. During this time a total of 16,816 digital fin photos were taken. These were reduced to a catalogue of 4,546 quality images, representing 921 photographed encounters with sharks. Individual basking sharks ranged from 2–10 m in overall length, and included both sexes. In the event relatively few sharks were encountered in the Firth of Clyde, and population estimates have therefore focused on the Inner Hebrides study area (Isles of Mull, Coll and Tiree).

**Table 2:** Results of boat-based surveys of basking sharks in the two study areas.

	Firth of Clyde			Inner Hebrides			Areas Combined	
Year	Distance (km)	Time (h)	Individual Sharks Photo-ID	Distance (km)	Time (h)	Individual Sharks Photo-ID	Individual Sharks Photo-ID	Number of Re-sights
**2004**	601	96	1	138.5	30	0	1	0
**2005**	1123.5	181	12	226	49	20	32	3
**2006**	406.5	54	1	226.5	39	27	28	0
**2007**	299.5	32	1	646	82	143	144	3
**2008**	440	38	0	685	71	239	239	10
**2009**	64	11.5	0	343.5	48.5	16	16	0
**2010**	125	19	0	507.5	95	231	231	10
**2011**	0	0	0	400	71	177	177	27
**Totals**	3059.5	431.5	15	3173	486	853	868	53

Shown are the distances covered (km) and duration (h) of dedicated boat-based surveys of the Firth of Clyde and Inner Hebrides study areas over the period 2004–2011, together with the numbers of separate individual basking sharks (of all fin grades) photographed and catalogued (Photo-ID) each year and the numbers of re-sightings (Re-sights). All re-sightings occurred in the Inner Hebrides. Note that the numbers of separate sharks photographed does not include re-sightings. The total number of photographed encounters is obtained by summing the number of sharks photographed and the number of re-sights. The total number of sharks recorded during surveys was higher again, since many of the sharks encountered could not be photographed in the available time.

### Numbers of Fins of Different Grades

Arising from the above total of 921 photographed encounters, 710 individual basking sharks were assigned to grades A1 –B2 (and thus considered to be individually identifiable), while 158 encounters were with grade C sharks (considered unlikely to be re-identifiable) (Table 2). The remaining 53 encounters were re-sightings of 41 previously recorded individuals.

While only a small proportion of dorsal fins were observed to be highly distinctive, upon close inspection of the images it was found that the great majority had features, such as injuries, marks or patterning, which could be used to distinguish individuals. 5.8% of fins were classified as grade A1, 15.9% as grade A2, 25.6% as grade B1, and 34.6% as grade B2, with only 18.2% being classified as grade C. Of the fins graded A1 to B2, over 90% showed some type of acquired scar, welt, or injury to the fin surface or edge, while 77% displayed distinct patterns of dark pigmentation on the sides.

### Re-sightings

**Fig 4:**
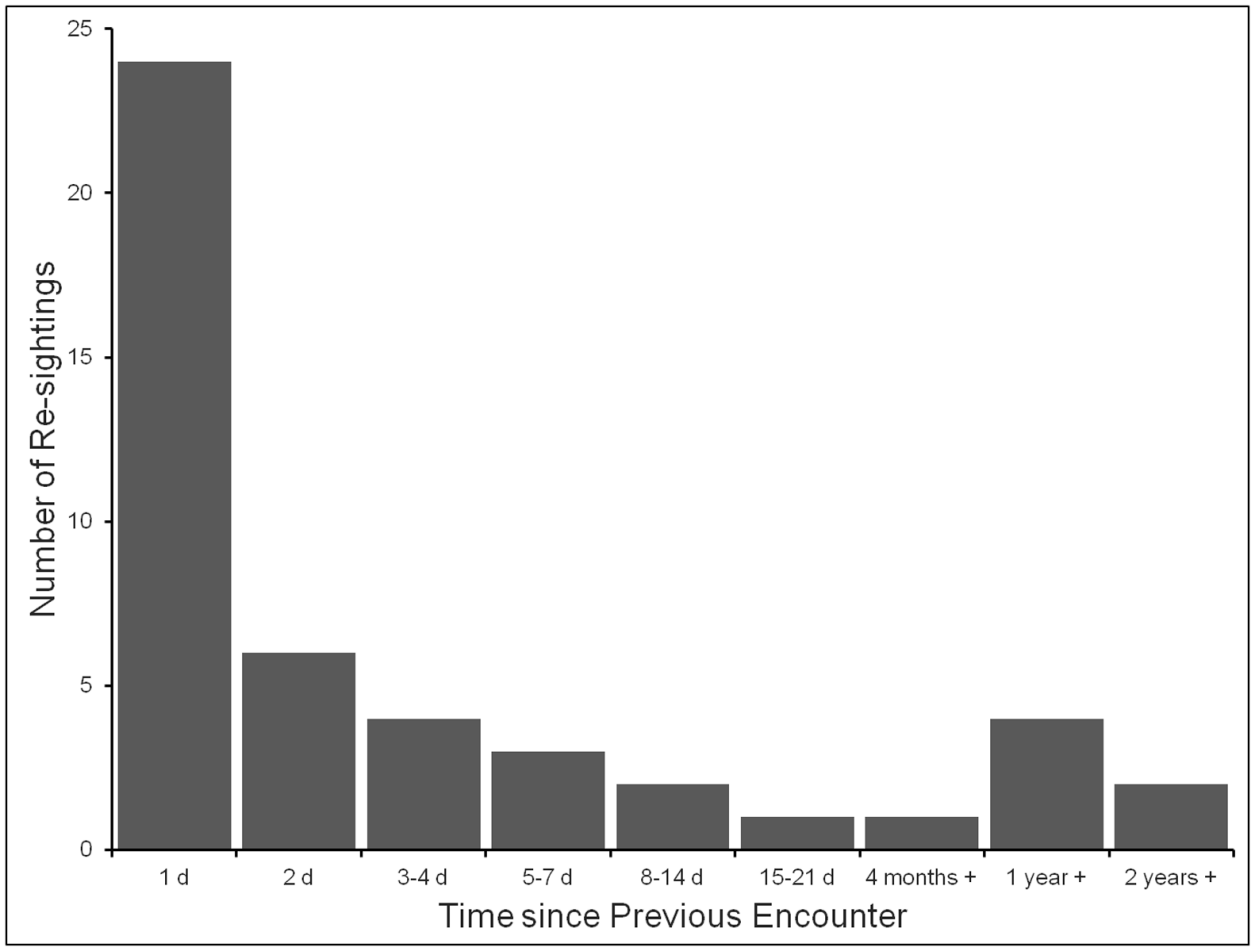
Numbers of basking sharks re-sighted after different periods of time following first encounter. The histogram shows the numbers of re-sightings of sharks after different periods of time, where the numbers of sharks re-sighted refer to the numbers of re-sightings and not the numbers of individual sharks involved, which were lower due to multiple re-sightings. The periods of time refer to the numbers of calendar days or weeks or years that had elapsed since the previous sighting; thus “1 day” means the shark was re-sighted on the following day, but not necessarily after more than 24 hours. All re-sightings were in the Inner Hebrides study area.

As stated above, 41 separate individuals were re-sighted. Of these nine were re-sighted twice and three re-sighted three times, giving a total of 53 re-sightings. Not included in the above data were 37 re-recordings that occurred within the 2h cut-off period. Thereafter the rate of re-sightings declined sharply, with six re-sightings later on the same day, and 41 during the period 1 to 125 d (Fig 4). There were six re-sightings thereafter, four in 2008 following initial encounters in 2007, and two in 2010 following initial recording in 2008.

This temporal pattern of re-sightings is as would be expected if individuals tended within a few weeks to move beyond the 50 km diameter study area. Most re-sightings occurred in fairly close proximity to the initial encounter, with a maximum distance between match locations of 17 km (Table 3). In part this reflects the limited size of the area which could be re-surveyed on a regular basis. All matches occurred within the Inner Hebrides study area, where the great majority of photographed encounters took place. There were no re-sightings in the Firth of Clyde or recorded movements between the two study areas. It was notable that four of the six inter-annual matches involved re-sightings within a few days of the calendar date of the initial encounter and at a relatively close location. (Since fieldwork took place annually within the same 4–6 weeks of the year, this might be a coincidence, but the possibility that some sharks establish regular migration routes and timings may be worth further consideration.)

**Table 3:** Re-sightings of basking sharks across years.

Date of Initial Sighting	Date of Re-sighting	Interval (days)	Distance (km)	Identification Characteristics	Fin Grade	Type of feature	Place on Fin
08/08/2007	07/08/2008	361	4.3	apex round	A1	natural	edge
				leading edge curved		natural	edge
				large notch in trailing edge		damage	edge
10/08/2007	10/08/2008	365	8	bump on apex	A2	damage	edge
				trailing edge crenulated		damage	edge
				pattern of crease scars on right side		natural	surface
11/08/2007	12/08/2008	364	3.6	apex round	A1	natural	edge
				notch top of trailing edge		damage	edge
				leading edge curved		natural	edge
				trailing edge smooth		natural	edge
25/08/2007	22/08/2008	362	17	apex round	A2	natural	edge
				bump on leading edge		damage	edge
				trailing edge crenulated		damage	edge
				leading edge curved		natural	edge
				lamprey mark		damage	surface
03/08/2008	26/08/2010	739	4.3	wide notch in apex	B2	damage	edge
				bump on leading edge		damage	edge
				trailing edge crenulated		damage	edge
03/08/2008	30/07/2010	704	1.9	apex flat, curled right	B1	natural	apex
				leading edge curved			
				trailing edge smooth			

The table shows for the six sharks re-sighted in subsequent years, the time intervals between initial sighting and re-sighting and the distance between the locations of first sighting and re-sighting. Also shown are the features that allowed individual identification of these sharks, whether the features were classified as natural features or the result of damage, and the grade of distinctiveness assigned to the fin on first cataloguing.

The 2011 season had the most matches, with 18 sharks involved in 27 re-sightings. The 2010 season produced the next highest number of re-sightings, with eight intra-seasonal matches. The proportion of recognisable individuals that were first recorded and then re-sighted within the same year ranged from 2.1% in 2007 to 10.2% in 2011 (excluding 2004, 2006 and 2009, in each of which < 30 animals were recorded). This range reflects marked variation in the numbers of basking shark surface feeding during given periods in different years, principally as a consequence of inter-annual variation in weather and sea conditions which influence the amount of food accumulated at the top of the water column and so available for surface feeding.

Of the sharks re-sighted during the same season (within periods of up to 125 days) 24.3% were grade A1, 21.6% grade A2, 27.0% grade B1, 16.2% grade B2, and 10.8% grade C (the latter despite the expectation of not being able to re-identify grade C individuals). There was thus little tendency for only higher grade sharks to be re-identified, which would have suggested that lower grade sharks were being overlooked. The mean number of distinctive features present, on which individual identification could be based, was 3.82 (range 1–10) on first sighting, and 4.00 (range 1–10) on re-sighting. Of the 41 sharks, seven appeared to have gained either one or two features: in four cases however this was because pigmentation marks were visible on the second occasion which had not been visible on the first, as a consequence of better illumination on the second occasion; otherwise one shark had acquired an extra scar (additional to some pre-existing ones), one had acquired an extra patch of epizoic copepods or barnacles on one side of the dorsal fin, and one had acquired both an extra patch of epizoa and a new lamprey scar. Conversely two sharks appeared to have lost a feature: in one case a patch of epizoic copepods appeared to have been permanently lost off one side of the fin, but in the second case it was found that another cluster of epizoa was positioned too low on the fin to be recorded on the second occasion. Thus all save one individual were characterised by more than one type of feature (injuries, scars, mottled patterning, epizoic barnacles etc.) and in all save one case (where some epizoic copepods had been lost) these features remained unchanged on re-sighting later within the same season.

**Fig 5:**
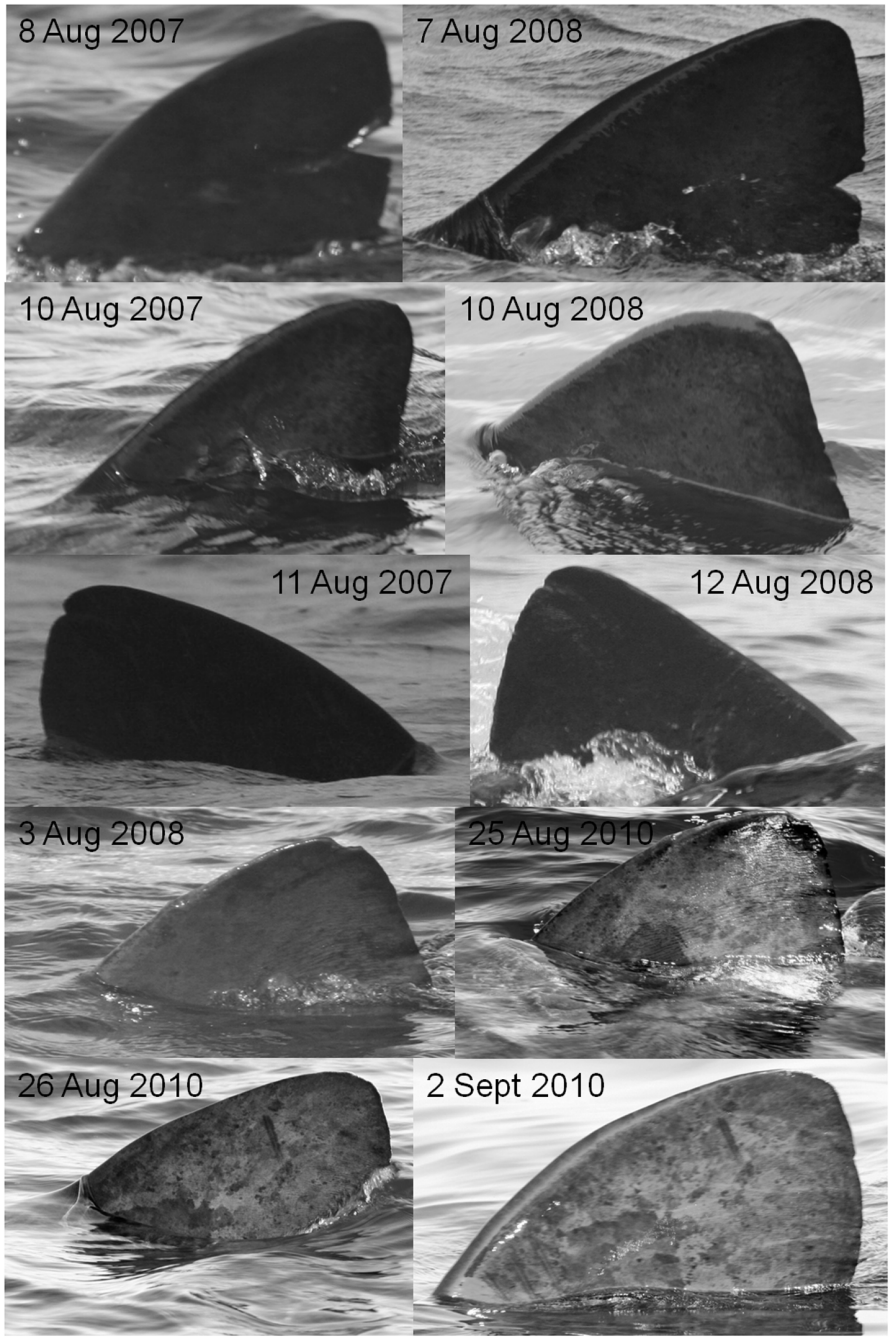
Examples of fins re-photographed after periods of one week to two years. The top three pairs of images show the same individuals photographed on first encounter and again after a period of one year, the fourth pair a shark resighted after two years, and the fifth pair a shark resighted after one week. The dates on which each photograph was taken are shown on the image.

Likewise comparison of the first and second sets of images obtained from sharks re-sighted after a year or more indicated that there had been relatively little change to distinguishing features. Table 3 shows the details of these sightings and lists the features by which the individuals were recognised. Two of these sharks were graded as A2, one as B1, two as B2, and one as grade C, indicating again no tendency for only high grade sharks to be re-sighted, even after a year or more. Comparison of images of the same fin taken on first encounter with those taken on subsequent re-encounter after periods of up to two years (Fig 5) indicates that crease-like scars and lamprey marks, as well both large and small notches, remain evident for at least a year, while raised scars (bumps) and patterns of trailing edge damage can be recognised after two years.

### Population estimates

The low proportion of previously recorded individuals re-encountered during the study suggests that the effective size of the population to which these sharks belong must be many times the total number of sharks that were catalogued. As already indicated, the number of between-year re-sightings was too low for any reasonably accurate estimate of regional population size (for example for the entire west of Scotland) to be generated from application of mark-recapture models, as had been intended. However, during short periods in 2010 and 2011, there were sufficient within-year re-sightings for us to be able to apply these methods to the much smaller Inner Hebrides study area, so deriving estimates for the local population temporarily present within this smaller area.

### Open-population estimates

Before applying any open-population models, RELEASE goodness-of-fit tests were applied to the data, including for the short (6–9 days) peak-sighting periods of 2010 and 2011. The tests indicated that for the 2010 peak-sighting period of 25 Aug– 2 Sept the number of re-sightings was too low to apply an open-population model with confidence. However for the 2011 peak-sighting period of 22–27 Aug RELEASE goodness-of-fit Tests 2 and 3 indicated that a general time-dependent Cormack-Jolly-Seber model did not violate the assumptions of equal capture probability and apparent survival (pooled χ^2^ = 5.78, df = 8, P = 0.67), with a variance-inflation factor (*ĉ*) of 0.72 not deviating substantially from the 1.0 *ĉ* value of the ideal-fit model [37]. In consequence, four permutations of the general Jolly-Seber open-population model were performed using the data for this short period and the parameter values and AICc weights shown in Table 4.

**Table 4:** Variants of the Jolly-Seber model used in population estimations.

Model	phi	p	pent	AICc	ΔAIC	AICc weight	*n*p
**phi(.)p(.)pent(t)**	0.62	0.25	0.06–0.29	171.95	0	0.706	8
**phi(.)p(t)pent(t)**	0.74	0.08–0.33	0.24–0.53	173.98	2.035	0.255	12
**phi(t)p(.)pent(t)**	0.31–0.83	0.23	0.12–0.26	178.16	6.216	0.032	12
**phi(t)p(t)pent(t)**	0.59–0.85	0.14–0.40	0.03–0.56	181.12	9.173	0.007	14

Parameter values and AICc weights for Jolly-Seber model variants used to estimate the temporary local population of the Inner Hebrides study area during the peak-sighting period of 22–27 Aug 2011: p = probability of capture, phi = probability of survival, pent = probability of entry into the study population, AICc = Akaike Information Criterion corrected for small sample sizes, *n*p = number of parameters used.

These results generated the local population estimates shown in Table 5. The model with the best fit to the dataset (AICc weight 0.71) was phi(.)p(.)pent(t), in which apparent survival and capture probabilities remained constant over the six-day sample period. The phi(.)p(t)pent(t) model also demonstrated a fairly good fit (AICc weight 0.26) and combined these two models have 97% support in the data. In view of this, model-averaging based on AICc weights was used to produce a point estimate of 202 recognisable basking sharks (95% CI = 109–297). Adjusting this estimate for the proportion of unrecognisable fins (that is, dividing by *θ* to allow for any unrecognisable C grade fins) yielded a total abundance estimate of 213 sharks (95% CI = 111–317) available to enter the sample area over the six-day period.

**Table 5:** Jolly-Seber model estimates.

	Recognisable Population				Total Population			
Model	*n*	*N*	SE	95% CI	*θ*	*N*_*tot*_	SE	95% CI
**phi(.)p(.)pent(t)**	111	194	36.64	142–292	.95	204	38.78	149–308
**phi(.)p(t)pent(t)**	111	228	65.32	145–420	.95	240	68.96	152–443
**phi(t)p(.)pent(t)**	111	191	36.11	140–287	.95	201	38.25	147–303
**phi(t)p(t)pent(t)**	111	191	46.76	131–326	.95	201	49.40	138–344
**model-averaged**	111	202	48.05	109–297	.95	213	52.75	111–317

The table shows the estimates of the total population temporarily present in the Inner Hebrides study area during the peak-sightings period 22^nd^–27^th^ August 2011 obtained using the different variants of the Jolly-Seber model indicated, together with the derived values of the following variables: *n* = total number of sharks encountered (including grade C), *N* = estimated recognisable population, SE = standard error, CI = confidence intervals, *θ* = proportion of recognisable sharks during sample period, *N*_*tot*_ = estimated total local population.

### Closed-population estimates

**Fig 6:**
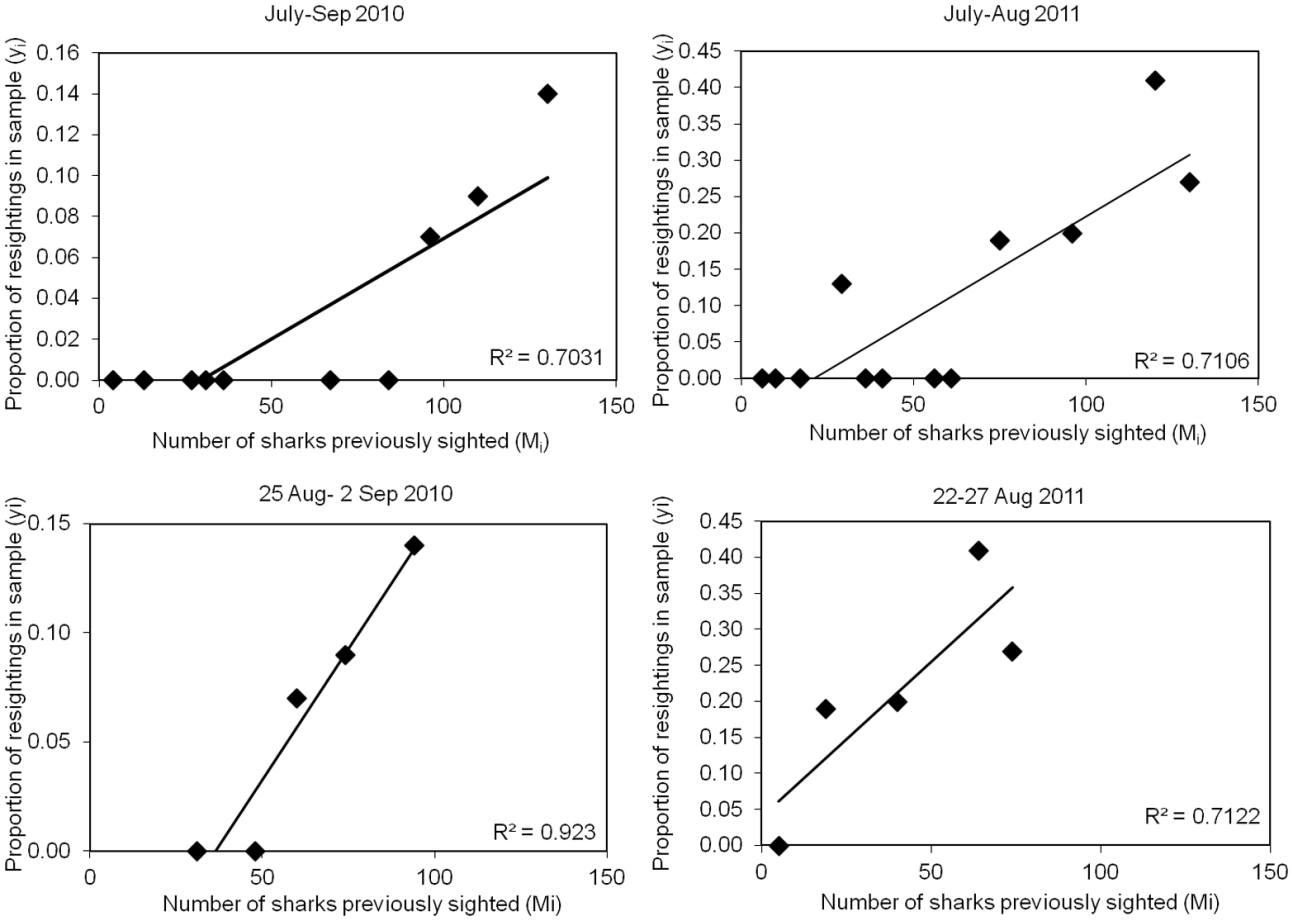
Increase in proportions of sharks re-sighted during the periods used for population estimates. The figure shows for the Inner Hebrides study area the increase with time in the proportions of recognisable sharks that were re-sighted plotted against the cumulative numbers of individuals previously recorded for: the entire 2010 season (July–September) (upper left), the 2010 peak-sightings period (August 25 –September 2) (lower left), the entire 2011 season (July–August) (upper right), and the 2010 peak-sightings period (August 25 –September 2) (lower right).

The two separate closed population models, Schnabel’s binomial model and the Schumacher & Eschmeyer model, were applied to the data from both 2010 and 2011, separately for both the peak sightings periods and for the whole season. As an indication of the degree of closure of the populations, in each case the proportion of sharks in each survey that were re-sightings of sharks recorded in earlier surveys (*y*_*i*_) was plotted against cumulative number of previously marked (photographed and recognisable) individuals present in the population immediately before each sample (*M*_*i*_) (Fig 6). The good linear fit for the peak-sighting period in 2010 (R^2^ = 0.923) as compared to the whole season for 2010 or either peak-sighting or whole season periods for 2011 indicates that this short sampling period adheres more closely to the assumptions of population closure and equal catchability than do the others.

The two methods generated very comparable population estimates (Table 6), but with the Schumacher & Eschmeyer method indicating lower standard errors. The Schumacher & Eschmeyer total population estimates were 1629 for the 2010 season and 459 for the 2011 season, and 985 for the 2010 peak sightings period (August 25^th^-September 2^nd^), and 201 for the 2011 peak sightings period (August 22^nd^-27^th^).

**Table 6:** Closed-population estimates.

Sample Period	Model	*n*	*r*	*θ*	*N*_*tot*_	SE	95% CI
**2010 Season**	Schnabel's binomial	202	7	0.80	1758	723.3	783–4355
	Schumacher & Eschmeyer	202	7	0.80	1629	291.9	1156–2724
**2010 Aug 25–Sept 2**	Schnabel's binomial	154	7	0.80	965	397.4	430–2390
	Schumacher & Eschmeyer	154	7	0.80	985	175.0	494–1683
**2011 Season**	Schnabel's binomial	169	22	0.95	493	108.1	318–781
	Schumacher & Eschmeyer	169	22	0.95	459	59.6	357–642
**2011 Aug 22–27**	Schnabel's binomial	111	21	0.95	180	40.4	114–289
	Schumacher & Eschmeyer	111	21	0.95	201	29.5	143–340

The table shows the estimates for the local populations of basking shark temporarily present in the Inner Hebrides study area during different periods in 2010 and 2011, together with the values of the following variables: *n* = shark encounters included, *r* = re-sights, *θ* = proportion of recognizable sharks in population, *N*_*tot*_ = total estimated abundance during sample period, SE = standard error, CI = confidence interval.

**Fig 7:**
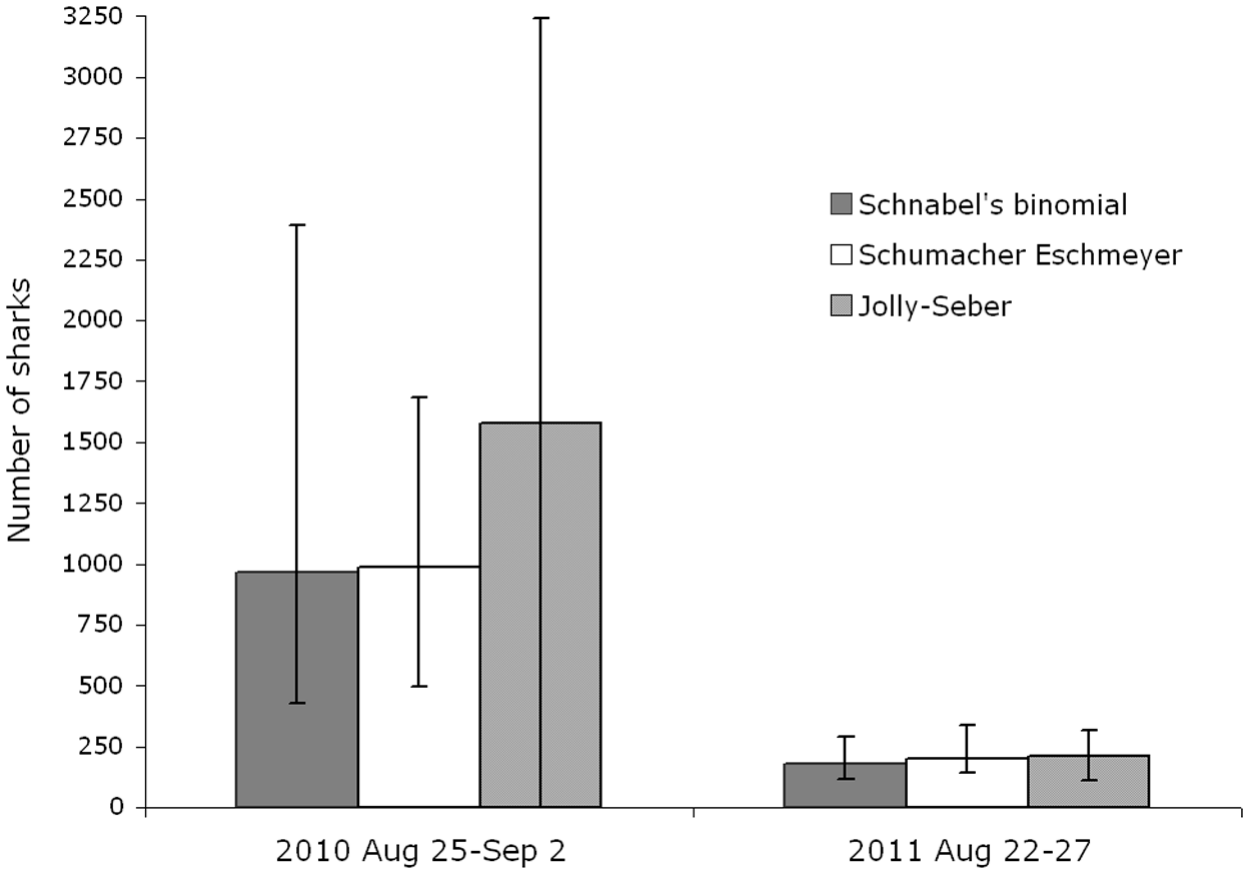
Comparison of abundance estimates obtained during peak periods in 2010 and 2011 for Inner Hebrides study area. The columns relate to the outputs of the open- and closed-population models indicated; the error bars represent 95% Confidence Intervals.

For both these peak-sighting periods, comparison of closed-population estimates with open-population estimates (Fig 7) shows that the two forms of model produced quite similar results (even though as mentioned above the confidence interval for the Jolly-Seber estimate for 2010 was too high for the estimate to be otherwise usable). These similarities might be expected given the short time-period covered by these estimates in relation to the 50 km diameter of the area being surveyed, as a result of which only a small proportion of sharks present may have left or entered the study area in the time concerned.

## Discussion

### Photo-identification

This study demonstrates the feasibility of using photo-identification to recognise individual basking sharks. 83% of photographed sharks bore distinctive dorsal fin features that would allow them to be recognised over a period of time. Obtaining high resolution images from a reasonably close distance of fins when they were well-illuminated proved to be crucial for this purpose. Although patience was required, such high quality images were not difficult to obtain since surface-feeding basking sharks frequently swam in close proximity to the research vessel, with no apparent disruption of feeding behaviour. In many cases individuals also displayed distinctive caudal injuries or body scars which were recorded and could be used to verify identification.

A number of studies have used semi-automated image recognition processing for comparing dorsal fins in marine mammals [45] [46] and it was anticipated that this would prove a useful tool for the large fins of basking sharks. However, this type of software depends primarily on the string length describing the outline of the full extent of the fin, whereas in our images of basking sharks, because of passing waves, a varying proportion (up to about a third) of the fin may be hidden by water. Finscan also ignores the surface patterning present on most fins. By contrast, visual inspection of fin images permits surface patterns to be compared and allowance made for occasions when part of the fin remains invisible.

In some other studies of very large sharks visual characteristics have been shown to provide a more reliable method of individual identification than conventional tagging. Graham & Roberts [19] used both photo-identification and two types of tags to mark individual whale sharks in a study off Belize. Re-sightings after both one and two years showed that more individuals were re-sighted by photo-identification than by tagging methods, indicating a significant rate of tag loss. The fact that some individual basking shark fins lack distinguishing features might be anticipated as an insuperable problem for our method, but the interpolation system used in this study makes allowance for the proportion of unrecognisable individuals. A similar method has been applied to small cetacean species [40], in which individuals are primarily identified by accumulated injuries to the dorsal fin trailing edge and surface [47] [48], but a proportion of individuals lack distinguishing marks.

The result that most of the re-sightings of individuals occurred within 10 days of their initial encounter is open to two explanations: either that the sharks lose their distinguishing features after this period of time, or that the sharks tend to move out of an area of the size of our Hebrides study area after about this length of time. The analysis of the images of the sharks that were re-encountered during a season supports the view that during this period few if any distinguishing marks are lost and recognition of previously encountered individuals can be undertaken with a fair degree of accuracy. Additionally, the six inter-annual matches presented support the view that most of the features used to make identifications remain relatively unchanged over longer periods of time. Comparison of the sets of images showed that pigmentation marks and healed scars persisted from one year to the next, as did a number of surface and edge features, including a small apex bump, a series of horizontal crease-like surface scars and a large trailing-edge notch (Table 3, App. 2). Contrary to our expectation, there was no obvious tendency for either short-term or long-term re-sightings to be mainly of animals graded as having the most distinctive fins (i.e. grades A1 or A2). A long-term photo-identification study of white sharks (*Carcharodon carcharias*) has shown similarly that individual trailing edge injuries can remain recognisable for over two decades [24]. Work on whale sharks (*Rhincodon typus*) has also demonstrated the persistence of visible scars and injuries for more than a decade [16]. The matches found in this study suggest that basking sharks may have a similar retention rate of identifiable fin characteristics as these other large elasmobranch species.

### Re-sightings and abundance estimates

Over the eight-year study period, 710 basking sharks were graded (A1 –B2) as individually recognisable, yet only 41 (4.7%) individuals were re-sighted, of which only six were re-sighted across years. Given the observed longevity of the fin features used to distinguish individuals, the results indicate that the basking sharks encountered in our study area must be drawn from a relatively mobile and much larger regional super-population. The spatial pattern of these re-sightings indicates that while the locations and timings of aggregations of basking sharks that occur at specific locations off the west coast of Scotland may be relatively predictable, they comprise individuals that change over periods of from one to several days. Similarly, the rate of decline in re-sightings against time suggests that the movements of the sharks tends to take them out of our larger (50 km diam) Hebrides study area after periods of one to two weeks.

These findings offer a contrast with photo-identification studies of the other well-studied planktivorous shark, the whale shark, in which photo-identification is normally based on the spot pattern within a portion of the flank photographed from underwater. Graham & Roberts [19] studying whale sharks in Gladden Spit (area *ca*. 73 km^2^) over five years used 571 images resulting in 123 individuals identified. Eight (44.4%) of 18 whale sharks were re-sighted after one year, 4 (22.2%) after two years and a further two sharks were re-sighted after 5 years. Off Holbox Island, Mexico, Ramírez-Macías et al. [33] identified 350 whale sharks from 1,184 photos taken over a four year period in an area of approximately 1,450 km^2^. They re-sighted 46 of these sharks after a year and 12 after two or more years, with re-sightings usually occurring within a few days or weeks. Using an open population JS model, they estimated a super-population size of 521–809 (95% CI, SE = 71) sharks at the aggregation site. In an approximately 211 km^2^ area around Mahe, Seychelles, Rowat et al. recognised 512 individuals over seven years, based on 1,149 photographs, and using an open population model generated a population estimate of 348 to 488 individuals [17]. Also Meekan et al. [16] found that 581 photographs taken within the 5,005 km^2^ area of Ningaloo Reef Western Australia led to the recognition of 159 individuals over 13 years, generating effective population estimates of 320–440 using an open-population model, and 300–500 using a closed-population model [16].

In a comparable photo-identification study of great white sharks [25] that aggregate (to feed on breeding seals) off Guadalupe Island, Mexico, 113 individuals were identified from above water dorsal fin images over nine years. Analysis using an open-population model estimated a pool of approximately 120 individuals visiting the site [25]. In this study and those on whale sharks, reliable estimates could be generated because a limited population of sharks appear to make use of each the study site on an annual basis. In the present study it appears that a larger population is passing through our study areas on a much less predictable basis, so that to date statistically reliable population estimates can only be generated for the numbers of sharks making use of our Inner Hebrides study area for 6–9 day-long peak-sighting periods. Data for these limited periods gave estimates for the size of the temporary loose aggregation present in our Inner Hebrides study area as 985 (95% CI, SE = 175) in 2010 (closed model), and 201 (95% CI, SE = 29.5; closed model) to 213 (95% CI, SE = 52.8; open model) in 2011.

Both whale sharks and white sharks appear to return more regularly and stay for longer within specific seasonal feeding areas compared to basking sharks, which appear to move continually between sites dispersed over broader-scale summer feeding grounds. A key difference between the ecology of basking sharks and that of the other two species is that white sharks (when around seal colonies) and whale sharks (when exploiting seasonal fish or coral spawning areas) appear to be using discrete feeding localities present within wider areas of relatively unproductive (tropical) ocean and over extended periods [49]. By contrast basking sharks inhabit the relatively plankton-rich waters of temperate latitudes, where favourable feeding zones may extend over large portions of the coast and the continental shelf [4]. Hence basking sharks are not dependent on such restricted feeding locations, but instead move from area to area on a time-scale of days and spatial scale of tens of kilometres. That basking sharks behave in this way is supported not only by the rapid decline in the frequency of re-sightings within our study area after only a few days, but by the results of tagging a series of basking sharks with pop-up satellite tags. These tags revealed that individuals typically travelled over large portions of the West of Scotland and adjacent continental shelf within the space of just a few weeks or months ([6] & Gore et al. unpublished data).

It was notable that some of the basking sharks re-sighted in subsequent years returned to the same area and did so very close to the anniversary of the dates on which they had originally been recorded. This suggests that at least some individuals may return more or less annually at about the same time of year to the sites of feeding aggregations that they have used before (Table 3). This proportion may however be markedly higher than we have recorded because, as our PAT satellite data have shown (Gore et al. unpublished data), most basking sharks spend only a small proportion of their time feeding at the surface. Hence not only may the numbers present in a limited area be much higher than those visible from a boat, but previously catalogued sharks may pass through an area without being detected.

### Limitations on mark-recapture analysis

The most significant obstacle to obtaining reliable mark-recapture estimates for either short-term local aggregations or wider regional super-populations was the very low rate of re-sightings. An open-population Jolly-Seber model requires *m*_*i*_ (number of previously marked individuals captured at occasion *i*) and *r*_*i*_ (number marked at occasion *i* and captured again later) of at least 10 per sample to generate fully reliable abundance estimates [35]. This difficulty has prevented or delayed mark-recapture analysis in some studies of large or highly migratory populations that yield few re-sightings [19]. A potential way to enhance the number of re-sightings for future mark-recapture modelling is to increase the volume of quality photographs available for analysis. Public involvement may offer a cost-effective way to increase the quantity of fin photos collected each year. The Shark Trust, Marine Conservation International and Save Our Seas Foundation have recently published a booklet encouraging wildlife tour vessel operators and private boaters to take quality dorsal fin photos and submit them for the benefit of photo-identification studies [31]. Similar collaborations between researchers and participants in the whale shark ecotourism industry have generated a global online database of whale shark photos that has been used for a number of mark-recapture studies [50]. In addition to providing valuable photographic data, this type of outreach allows members of the public to become better informed and participate in shark research and conservation.

### Implications for basking shark conservation and management

The abundance estimates produced by this study, of 985 in 2010 and 201 or 213 in 2011, related to basking sharks temporarily present in a limited study area that encompassed the west of the islands of Mull, and the islands of Coll and Tiree. These numbers may seem high for a limited area, compared to the modest numbers of sharks typically encountered feeding at the surface. However, as already emphasised, the numbers of sharks observed on the surface may be much smaller than those actually present, since, as our studies indicate, on average these sharks spend only about 10% of their time feeding at the surface (Gore et al. unpublished data). Nevertheless, on several occasions over the last few years, a Coll-based fisherman has reported to us seeing in one day at least 200 animals surface feeding around this one island. Similarly, on three occasions during 2011 and 2012, we had independent reports from fishermen and wildlife boat operators, from a number of locations inside or within 25 km of our study area, of a combined total of approximately 1,000 sharks observed surface feeding during a single day. In addition, during recent surveys of an area extending from the island of Tiree southwards (undertaken by RPS Group plc in relation to a proposed offshore wind farm development) observers reported sighting 918 sharks in a single day (5^th^ August 2012). These sightings may represent occasions when conditions were most favourable to surface feeding, but they nevertheless indicate that our mark-recapture estimates for the peak numbers of sharks present in this limited study area are credible.

While we could generate population estimates for the numbers of basking sharks temporarily present over 6–9 d within our Inner Hebrides study area, we could not on this data alone generate a reliable estimate for the larger regional population that appears to make use annually of a much larger region covering at least the west coast of Scotland and adjacent continental shelf. This was because so few individuals were re-sighted in successive seasons. Notably, while 231 animals were photographed in 2010 and 177 in 2011, none of the individuals recorded in 2010 were re-sighted in 2011, even though 23.5% of fins photographed in those years were graded as A1 or A2. Comparable low re-sighting rates have been obtained for basking sharks tagged with conventional placard tags off the north and west coasts of Ireland (Berrow et al. unpublished data), lending credence to our values.

Within our study area, the percentage of sharks recorded in one year and re-sighted in the next have so far ranged from 0–2.8%, with a mean of 0.5%. We were concerned that these very low rates of re-sighting might primarily reflect a failure to recognise individual sharks that had been previously encountered. However the detail visible on the fins of the great majority of newly encountered sharks appears quite sufficient to determine with some confidence that these individuals had not been previously documented. Since the very low rates of re-sighting were against our own initial expectation, we both repeated the complete matching exercise and greatly extended the study period, but with the same result. Taken at their face value these very low rates of re-sighting suggest that the super-population of sharks making use of the wider region (the continental shelf of the West of Scotland and adjacent areas) must be considerably greater than the population present over short periods in our limited study area.

## Supporting Information

S1 Table Features used for cataloguing individual sharks. The table shows the database fields and information or options used to catalogue individual sharks, and subsequently used in the search for matches.
(PDF)
