# Supplementary material for: Use of Photo-Identification and Mark-Recapture Methodology to Assess Basking Shark (Cetorhinus maximus) Populations
Source: PLoS One. 2016 Mar 1;11(3):e0150160. doi: 10.1371/journal.pone.0150160 (PMC4773138; doi:10.1371/journal.pone.0150160)
Supplement: S1 Table — The table shows the database fields and information or options used to catalogue individual sharks, and subsequently used in the search for matches. (PDF) [file pone.0150160.s001.pdf]

**Database Field**	**Definitions and /or Options**
**Photographer**	Name of researcher taking photograph & holding original images
**File Reference Name & No.**	Name of original computer, drive & folder where images held
**Shark Catalogue Accession No.**	Unique record identifier within database
**Matched Reference / ID No.**	Identifiers of other entries to which individual matched
**Region**	Study area where recorded e.g. Inner Hebrides : Firth of Clyde
**Date**	Day, month & year
**Time**	Hour & minute
**Location**	Decimal latitude & longitude
**Size**	Total length in meters, to the nearest half meter
**Quality of Photographic Images**	Graded 1 – 3; 1 = high resolution, well lit, many details visible
**Grade of fin**	Distinctiveness of fins - A1 major marks or injuries, usually visible from a distance: A2 conspicuous marks or injuries : B1 – clear marks or injuries visible on close inspection: B2 - visible marks or injuries visible on detailed study : C - no evident marks or injuries
**Best Show**	Proportion of fin seen above water on best images: 30% : 60% : 90%
**Fin Shape**	General shape of fin profile – Standard (as in guide book): Pyramidal: Right Angled: Lobed: Conical: Tulip (broadening slightly above the base as in a tulip bud)
**Fin Apex**	Shape of apex of fin - Rounded: Pointed: Missing:: Flat : Other (other feature present at tip)
**Surface Right side**	Indicating any features on right flank of fin - Mark: Scar: Injury: Other Feature: No Feature: Unseen
**Surface Left side**	As for Right side
**Leading Edge Shape**	Shape of profile – Straight : Curved : Roman (as in roman-nosed)

**Leading Edge Feature**	Indicating any features on edge - Mark: Scar: Injury: Other Feature: No Feature, Unseen (also for scars & injuries - healing or fresh/raw)
**Trailing Edge Nature**	Whether trailing edge of fin - Smooth: Frayed: Crenulated
**Top -third Trailing Feature**	Features in top third of trailing edge - Nick: Notch: Large Notch (>10cm): Scrape: Cut: Rope burn: No Feature: Unseen: Other
**Mid-third Trailing Feature**	As for Top-third Trailing Feature
**Bottom-third Trailing Feature**	As for Top-third Trailing Feature
**Top Right Surface Feature**	Features on upper right flank of fin - Lamprey: Copepods: Welts: Roughness : Rope burn: Tyre marks: Scrapes: Dark speckles: Dark blotches: Dark patterning : No dark pigmentation
**Mid Right Surface Feature**	As for Top Right Surface
**Bottom Right Surface Feature**	As for Top Right Surface
**Top Left Surface Feature**	As for Top Right Surface
**Mid Left Surface Feature**	As for Top Right Surface
**Bottom Left Surface Feature**	As for Top Right Surface
**Placeholder Image Right**	Reference number of best image of right side
**Placeholder Image Left**	Reference number of best image of left side
**Good Images**	Reference numbers of other selected good images
**No. of Images**	Number of images retained in the database
**Comments**	Comments e.g. on distinctive features of caudal fin or body
